# Large-scale brain networks and intra-axial tumor surgery: a narrative review of functional mapping techniques, critical needs, and scientific opportunities

**DOI:** 10.3389/fnhum.2023.1170419

**Published:** 2023-07-13

**Authors:** Timothy F. Boerger, Peter Pahapill, Alissa M. Butts, Elsa Arocho-Quinones, Manoj Raghavan, Max O. Krucoff

**Affiliations:** ^1^Department of Neurosurgery, Medical College of Wisconsin, Milwaukee, WI, United States; ^2^Department of Neurology, Medical College of Wisconsin, Milwaukee, WI, United States; ^3^Mayo Clinic, Rochester, MN, United States; ^4^Department of Biomedical Engineering, Medical College of Wisconsin, Marquette University, Milwaukee, WI, United States

**Keywords:** connectivity, brain tumor, networks (circuits), fMRI, diffusion weighted imaging (DWI), magnetoencephalography (MEG), electrocorticography, direct cortical stimulation

## Abstract

In recent years, a paradigm shift in neuroscience has been occurring from “localizationism,” or the idea that the brain is organized into separately functioning modules, toward “connectomics,” or the idea that interconnected nodes form networks as the underlying substrates of behavior and thought. Accordingly, our understanding of mechanisms of neurological function, dysfunction, and recovery has evolved to include connections, disconnections, and reconnections. Brain tumors provide a unique opportunity to probe large-scale neural networks with focal and sometimes reversible lesions, allowing neuroscientists the unique opportunity to directly test newly formed hypotheses about underlying brain structural-functional relationships and network properties. Moreover, if a more complete model of neurological dysfunction is to be defined as a “disconnectome,” potential avenues for recovery might be mapped through a “reconnectome.” Such insight may open the door to novel therapeutic approaches where previous attempts have failed. In this review, we briefly delve into the most clinically relevant neural networks and brain mapping techniques, and we examine how they are being applied to modern neurosurgical brain tumor practices. We then explore how brain tumors might teach us more about mechanisms of global brain dysfunction and recovery through pre- and postoperative longitudinal connectomic and behavioral analyses.

## 1. Introduction

Traditionally, neurosurgical decision making has focused on preserving “eloquent” cortical nodes and their underlying white matter pathways (Burchiel et al., 1989). More recently, however, there is growing recognition that this view oversimplifies brain function as “modular,” when, in fact, each module is likely part of a semi-redundant network that processes information and generates behavior (Dadario and Sughrue, 2022; D’Angelo and Jirsa, 2022). Interest has also broadened from simply mapping movement and language to better understanding cognition and affective changes related to lesions and their surgeries (Dadario et al., 2023). As such, debates surrounding what defines truly “eloquent” brain have begun, and the line continues to shift with growing knowledge (Dadario and Sughrue, 2022). As brain network science advances, new connectivity atlases are being generated and models of associated functions are being built (Yeo et al., 2011; Peer et al., 2017; Schaefer et al., 2018; Dadi et al., 2020). Brain tumors provide a unique opportunity to probe these large-scale brain networks with focal and sometimes reversible lesions, allowing neuroscientists the unique opportunity to directly test hypotheses about underlying brain structural-functional relationships. Moreover, if a more complete model of neurological dysfunction is to be defined through disconnections, recovery of function might therefore be viewed as a series of required reconnections, whether through native or alternative networks (Krucoff et al., 2016). To this end, a better understanding of large-scale brain networks may aid in developing more efficacious therapies to alleviate otherwise difficult-to-treat functional deficits (Dadario et al., 2021). This review therefore begins with a brief introduction to several large-scale brain networks and the mapping techniques used to define them. We then discuss how these mapping techniques can impact surgical decision making for patients with brain tumors. Finally, we examine how brain tumors can uniquely advance our network-based understanding of neural impairments and recovery when these techniques are applied longitudinally with standardized behavioral testing.

## 2. Large scale brain networks

In this section, we provide a brief overview of the most surgically relevant large-scale brain networks: somatomotor, language, and cognitive networks. This section is intended for a reader who is largely unfamiliar with these concepts, and references to more in-depth literature reviews for each are provided.

### 2.1. Somatomotor network

Topographically, the somatomotor network is defined largely by the pre- and post-central gyri (i.e., peri-rolandic area) that execute movement and process sensation (Yeo et al., 2011). This network is commonly considered “eloquent” (Penfield et al., 1937; Burchiel et al., 1989) due to the potential for permanent and debilitating neurological deficits if injured (Kandel et al., 2012). Classically, its somatotopy has been defined as a stereotyped medial-to-lateral modular progression of body parts from distal lower extremity to face (Roux et al., 2018, 2020). However, newer connectomic-based discoveries suggest that previously unknown somatomotor association areas may exist between and modulate classical effector regions to facilitate whole-body motor planning (Gordon et al., 2023). The clinical significance of this finding is unknown, but the discovery of these interdigitated areas and its associated redefinition of the classical homunculus may reveal more neuroplastic potential for this network than previously recognized.

Clinically, the somatomotor region has demonstrated only minimal plastic potential in adults (Picart et al., 2019). Because of this, portions of tumors invading the precentral gyrus are sometimes inoperable, depending on the tumor type (Seidel et al., 2013). Unsurprisingly, reduced connectivity between homotopic pairs of cortical parcels within the somatomotor network is associated with reduced overall survival (Daniel et al., 2021), as is reduced structural connectivity between neighboring regions (Wei et al., 2022). Likewise, tumors invading the corticospinal tract in otherwise healthy individuals correlates with worse overall survival (Mickevicius et al., 2015). Therefore, an adaptation of lesion connectome mapping (i.e., “survival connectome mapping”) may provide valuable prognostic information.

Additionally, somatomotor structural and behavioral alterations associated with chemo and radiation therapies is being investigated (Seibert et al., 2017). While these therapies are known to result in decreased cortical thickness (i.e., gray matter loss), associated connectomic changes are newer targets of investigation (Seibert et al., 2017; Mitchell et al., 2020; Daniel et al., 2022; Zhu et al., 2022).

### 2.2. Language network

Unlike many of the canonical resting-state networks (i.e., somatomotor, default mode, dorsal attention, etc.), the language network is best described functionally based on various language tasks (Aabedi et al., 2022). Topographically, it contains the classic Broca’s and Wernicke’s areas with the arcuate fasciculus joining them, as well as their dorsal and ventral processing streams (Chang et al., 2015). The language network is also considered “eloquent” in that damage to its key components can result in permanent debilitating deficits. Unlike the somatomotor network, language areas appear more widely distributed and can be highly plastic under the right circumstances (Picart et al., 2019; Aabedi et al., 2022). This plasticity can involve increases in activation of the right hemisphere and cerebellum; however, language functions can also relocate to “atypical” regions of the frontal and temporal lobes ipsilesionally (Thiel et al., 2001). Interestingly, recent evidence suggests that language network plasticity may be facilitated to extend surgical resections by intracranial electrical stimulation and prehabilitation (Rivera-Rivera et al., 2017). For those interested in a more detailed review of language network plasticity, we refer the readers to a recent review (Pasquini et al., 2022).

### 2.3. Cognitive networks

**FIGURE 1:**
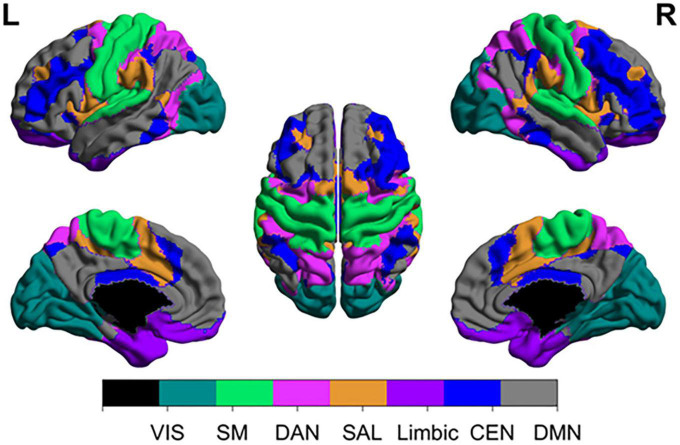
Anatomical topographies of canonical large-scale networks. Networks were generated from those reported in Schaefer et al. (2018) (available at: https://github.com/ThomasYeoLab/CBIG/tree/master/stable_projects/brain_parcellation/Schaefer2018_LocalGlobal) with updated terminology. VIS, visual; SM, somatomotor; DAN, dorsal attention network; SAL, salience; CEN, central executive network; DMN, default mode network.

While certain cortical processes (i.e., language and motor output) can be easily mapped intraoperatively with direct electrical stimulation (see section “3.1.1. Direct electrical stimulation”), more complex and abstract cognitive processes [i.e., working memory, attention, decision making, etc., (Buckner et al., 2008)] are more difficult to pinpoint. Consideration of such networks is critical as these networks may be directly affected in as much as 80% of patients with brain tumors (Morell et al., 2022). However, the difficulty in assessing them intraoperatively, difficulty in predicting long-term outcomes, and the unpredictable natural history of many postoperative cognitive deficits makes it uncommon to consider the networks in standard of care surgery (beyond avoiding operating on bilateral lesions). Table 1 represents a brief summary of the most well-known cognitive networks, their associated anatomical contributions, functions, and pathologies. Figure 1 provides a visual representation of these networks. For a more detailed review of these cognitive networks, we refer readers to the reviews and articles listed in the table.

**TABLE 1:** Summary of various cognitive networks.

Network (references)	Example anatomical areas	Examples of functions	Example of associated pathology
Default mode network (Buckner et al., 2008; Brakowski et al., 2017; Burks et al., 2017; Stephens et al., 2021)	● Posterior cingulate ● Precuneus ● Medial prefrontal ● Inferior parietal cortex	● Autobiographical memory ● Thinking of the future ● Theory of mind ● Moral decision making	● Autism spectrum disorders ● Schizophrenia ● Alzheimer’s disease ● Depression ● Butterfly glioblastoma
Salience a.k.a. ventral frontal parietal/attention/ventral attention/cingulo-opercular network (Uddin, 2015; Brakowski et al., 2017; Seeley, 2019; Uddin et al., 2019)	● Insula ● Anterior cingulate ● Frontal operculum	● Receives sensory (internal and external) information, distills, and exports to rest of brain ● Mediator between active and passive states of mind	● Autism ● Schizophrenia ● Frontotemporal dementia ● Depression
Central executive a.k.a. frontoparietal/executive control network (Smith et al., 2009; Yeo et al., 2011; Brakowski et al., 2017; Kim, 2019; Uddin et al., 2019; Witt et al., 2021)	● Dorsolateral prefrontal cortex ● Inferior parietal lobule	● Working memory (especially maintenance and retrieval) ● Cognitive control ● Action inhibition ● Language ● Executive control	● Aphasia ● Depression
Dorsal attention/dorsal frontoparietal network (Kandel et al., 2012; Ptak et al., 2017; Uddin et al., 2019; Sugiura et al., 2020; Witt et al., 2021)	● Frontal eye fields ● Superior parietal lobule	● Spatial attention ● Mental rotations ● Saccades ● Reaching ● Working memory	● Visual spatial neglect

The literature uses various names for several of these networks. Some work has begun to coalesce the nomenclature into a common terminology (see Uddin et al., 2019 and Witt et al., 2021). This table uses several of the various terms to facilitate broader clarity.

## 3. Brain mapping techniques

In this section, we review several commonly used brain mapping techniques and their applications to network neuroscience. We examine brain stimulation, electromagnetic recording, and magnetic resonance imaging techniques and how they have been used to probe large networks.

### 3.1. Brain stimulation

#### 3.1.1. Direct electrical stimulation

Direct electrical stimulation (DES) of the cortex and white matter gained popularity through the work of Penfield et al. (1937) in the 1930s. This technique involves applying an electrical stimulus to a cortical or subcortical surface and measuring the downstream response (Aaronson et al., 2021). In the primary motor area, for example, motor evoked potentials (MEPs) or gross muscle twitches are observed in response to a stimulus (Viganò et al., 2019). In speech and language areas, perturbations of language tasks are seen when critical sites are stimulated (Lu et al., 2021). Stimulation is frequently applied at 50–60 Hz (Europe vs. North America, respectively) for bipolar stimulation and 5–10 pulse bursts at 250–500 Hz for monopolar stimulation, although optimal stimulation parameters have never been formally established (Aaronson et al., 2021). When using monopolar stimulation, anodal (i.e., positive-current) is used for the cortex, whereas cathodal (i.e., negative-current) stimulation is used for the white matter (Seidel et al., 2013).

**FIGURE 2:**
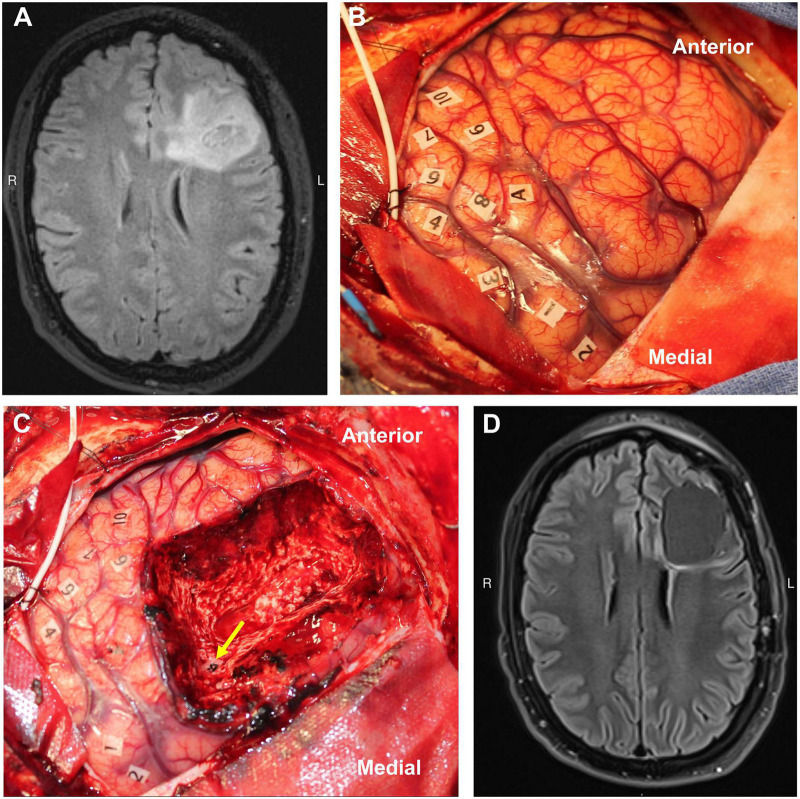
Example of direct cortical and white matter stimulation mapping during an awake craniotomy performed by the senior author. The numbers represent areas of positive motor evoked potentials in the precentral gyrus (i.e., the motor homunculus), and the letters represent area of speech disturbance during stimulation in the middle frontal gyrus **(A)** and superior longitudinal fasciculus **(B)**. Panels **A/C**–preresection, **B/D**–postresection [MRIs are preop **(A)** and 3-mo postop **(D)** FLAIRs]. Used with permission from Harwick et al. (2023) all rights retained.

Direct electrical stimulation (DES) is an invaluable surgical tool, and it is the gold standard technique for identifying critical functional anatomy (Figure 2) intraoperatively during brain tumor surgery. In contrast to techniques like fMRI, DES provides direct information about the necessity of a local brain region in completing a task, and its application is generally thought to mimic the effects of a temporary lesion in the stimulated area. Limitations to DES include the surgical exposure (i.e., it can only be applied in and around the surgical site) and limited intraoperative testing time during awake cases. Generally, there is no opportunity for bilateral DES. In addition, tasks are generally constrained to simple movements or verbal tasks that can be performed in the operating room during an awake craniotomy (Harwick et al., 2023). However, with the advancement of FDA-approved cranial implants for epilepsy that can both stimulate and record neural data, such opportunities to probe larger networks in more naturalistic environments is changing (Krucoff et al., 2021; Stangl et al., 2021). A further more detailed review of DES can be found here (Aaronson et al., 2021).

#### 3.1.2. Deep brain stimulation

**FIGURE 3:**
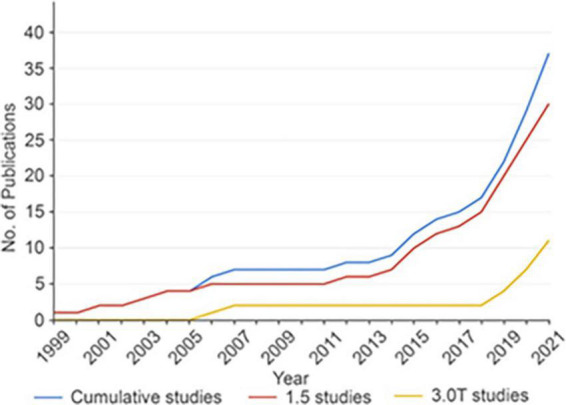
Cumulative number of fMRI DBS studies over time. The overall rate of DBS fMRI studies is increasing over time. The rate of all DBS fMRI studies is shown in blue, while studies performed with 1.5T MRI are shown in red and studies performed with 3T MRI are shown in yellow. DBS, deep brain stimulation; fMRI, functional magnetic resonance imaging; No., number; T, tesla. Reproduced with permission from Loh et al. (2022), all rights reserved.

For over 30 years, deep brain stimulation (DBS) has been use to compliment single-cell and field potential recordings in the identification, mapping, and characterization of subcortical structures for conditions such as tremor, Parkinson’s disease, and dystonia (Cooper et al., 1980; Lee et al., 2019; Figure 3). Intraoperative micro- and/or macro-stimulation techniques in awake DBS patients help identify somatotopy of the sensory-motor sub-regions of the ventralis oralis posterior, ventral intermedius, and ventral caudal thalamus, subthalamic nucleus (STN), and globus pallidus. Similarly, such stimulation techniques help optimize the localization of putative sub-cortical therapeutic targets by documenting maximal clinical benefit with minimal stimulation thresholds while mitigating untoward effects such as sustained paresthesia’s, contractions, diplopia, dysarthria or phosphenes associated with current spread to sub-cortical structures such as the medial lemniscus, internal capsule, third nerve fascicles and optic tracts [see Gross et al. (2006) for details].

Over the last several years, there has been a shift in focus from what is being stimulated at the local level as described above (i.e., ventral intermedius, ventral caudal, STN, global pallidus internus) toward assessing what is being engaged at the whole-brain, or network, level. This has been fueled by the growing understanding that DBS-treatable conditions (e.g., tremor, Parkinson’s disease, dystonia) represent different forms of brain circuitopathies or connectopathies. Recent connectomic DBS studies have examined the functional connectivity of stimulated brain regions and how these relate to patient outcomes [summarized in Loh et al. (2022)]. Techniques such as positron emission topography (PET), single-photon emission computerized tomography (SPECT), functional magnetic resonance imaging (fMRI), electroencephalography (EEG), and magnetoencephalography (MEG) have all been employed on patients with implanted DBS systems. For example, fMRI studies have shown that stimulation of the subthalamic nucleus (STN) in Parkinson’s disease patients is consistently associated with changes within the cortico-basal-ganglia-thalamo-cortical loop and cerebellum. Anecdotally, a pair of studies demonstrated normalization of functional connectivity in Parkinson’s disease patients toward healthy brain states with STN DBS (Saenger et al., 2017; Kahan et al., 2019). Ventral intermedius-DBS for essential tremor was associated with activation in the contralateral cerebellar cortex and deep cerebellar nuclei with the therapeutic effectiveness showing the strongest correlation with cerebellar activation (Gibson et al., 2016). Interestingly, stimulation of the periventricular gray matter (PVG) and ventral caudal nucleus of the thalamus in chronic pain patients seems to produce a different set of anatomical activation patterns in fMRI that correlate with the presence or absence of induced paresthesia’s (Rezai et al., 1999). Findings for further reading, readers are referred to the review by Loh et al. (2022).

#### 3.1.3. Cortico-cortical evoked potentials

Cortico-cortical evoked potentials (CCEP) and cortico-cortical spectral responses (CCSR) measure spread of current along white matter pathways in response to a directly administered cortical stimulus (Sala et al., 2021; Yamao et al., 2021). CCEPs and CCSRs are generated by applying electrical stimulation to the cortex through a pair of electrodes and recording from other electrodes proximal and/or distal to the stimulation site (Matsumoto et al., 2004). It is common to employ a train of ∼1 Hz stimulus of 20 or more stimuli to allow the peri-stimulus responses (∼ −100 to +500 ms) to be stacked and averaged (Matsumoto et al., 2004; Sala et al., 2021). The observed electrical flow is typically directional down axons, allowing a causal assessment of “effective connectivity” (Sala et al., 2021; Yamao et al., 2021).

**FIGURE 4:**
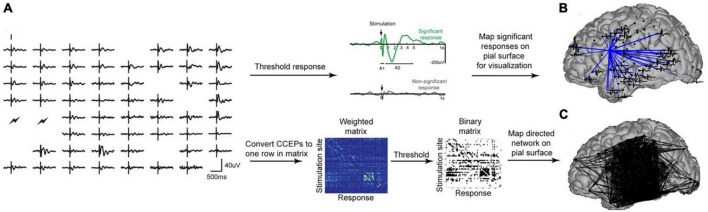
**(A)** Shows processing pipeline for CCEPs. **(B)** Shows significant connections from a single stimulation site. **(C)** Shows a weighted connectivity map. Figure reproduced with permission from Keller et al. (2014).

Cortico-cortical evoked potentials (CCEPs) have been used to assess the effective connectivity (Figure 4) of the language network across the superior longitudinal, inferior longitudinal, and arcuate fasciculi (Keller et al., 2014). Similarly, CCEPs have been used to map shorter-range connections through the frontal aslant tract, uncinate fasciculus, sensorimotor pathways, dorsal and ventral visual streams, and u-shaped fibers between adjacent gyri (Silverstein et al., 2020). Other studies have used CCEPs to map the outgoing effective connectivity of a number of other structures as well including the cingulate cortex (Oane et al., 2020).

Cortico-cortical evoked potentials (CCEPs) appear to be especially effective when combined with diffusion tractography as this allows assessments of not only strength of connectivity (mV) and latency (ms), but also velocity of electrical flow (m/s) (Silverstein et al., 2020). Historically, studies using this technique have been conducted with the patient awake either intraoperatively or in an epilepsy monitoring unit (Prime et al., 2018; Yamao et al., 2021). Some interest recently, however, has been in utilizing CCEPs intraoperatively for asleep language mapping (Yamao et al., 2014; Giampiccolo et al., 2021b).

Unique benefits to CCEPs include an ability to infer causal relationships between areas. CCEPs also appear to be less effected by edema than diffusion tractography-based measures of connectivity. Limitations to CCEPs and CCSRs largely revolve around surgical exposure, similarly to DES. Currently, there is minimal opportunity to investigate interhemispheric connections or connections between disparate areas of the brain intraoperatively. In epilepsy, however, stereo electroencephalography (sEEG) pre-resection is often used to map epileptogenic foci, and this can be done bilaterally (Rosenow and Menzler, 2013; Hagiwara et al., 2022). For a more detailed review of CCEPs techniques, we refer the reader to a recent review (Yamao et al., 2014).

#### 3.1.4. Transcranial magnetic stimulation

Transcranial magnetic stimulation (TMS) is a brain stimulation technique that has gained popularity due to its non-invasiveness and general safety. An electrical current is passed through a wire coil (typically in a figure-8 shape) that induces a magnetic field strongest under the intersection of the coils, generating a focal, targeted electrical current in the cortex (Salinas et al., 2007; Laakso et al., 2014). To mitigate the reduced spatial resolution of TMS relative to DES, stimulations are repeated using a grid or adaptive learning algorithm. TMS has gained recent interest for pre-resection mapping of motor and language areas (Sollmann et al., 2021; Schiavao et al., 2022). For motor mapping, the point eliciting the largest motor evoked potential, which has an error ∼2 mm relative to DES, is considered the primary motor area (Tarapore et al., 2012; Engelhardt et al., 2019). Limitations of TMS include a strong dependence on coil orientation with respect to the head and correct stimulation intensity and a limited depth of penetration (Sollmann et al., 2021; Schiavao et al., 2022; Weise et al., 2023). Strengths include causality of available inferences, non-invasiveness, temporary lesioning or faciliatory effects, and the ability to stimulate the contralateral cortex (Mirchandani et al., 2020; Ozdemir et al., 2021). For further reading see Sollmann et al. (2021).

### 3.2. Electromagnetic recording

In this section, we review several common electromagnetic recording techniques followed by an overall comparison. EEG, MEG, and electrocorticography (ECoG) capture signals that derive from the same underlying electrical activity of the cerebral cortex: ionic currents generated by summated excitatory and inhibitory post-synaptic potentials in the most superficial layers of the cerebral cortex.

#### 3.2.1. Electroencephalography

Electroencephalography (EEG) is recorded non-invasively through scalp electrodes. Strengths of EEG include non-invasiveness and high sampling rate relative to functional MRI. Weaknesses include low spatial sensitivity and poor ability to record at depth.

#### 3.2.2. Electrocorticography

Electrocorticography (ECoG) is recorded invasively through subdural, epidural, or depth (i.e., intraparenchymal) electrodes. While ECoG is highly spatially specific and provides excellent temporal resolution, it is invasive and suffers from limited spatial coverage.

#### 3.2.3. Magnetoencephalography (MEG)

Magnetoencephalography (MEG) records extremely weak magnetic fields of the order of femtoteslas that are generated by cortical neural activity. Specialized and expensive hardware and a magnetic shielded room to attenuate environmental electromagnetic noise are required to record magnetic activity of cerebral origin. MEG is non-invasive and highly spatially and temporally specific, but it is also relatively expensive and not available at many centers.

#### 3.2.4. Similarities and differences across electrical recording techniques

Electroencephalography (EEG), ECoG, and MEG can all resolve dynamics of cortical activity with millisecond resolution. Because of the folded geometry of the cerebral cortex, the orientations of electrical fields generated by different patches of cortex can differ. Consequently, there are differences in the neural activity best represented in EEG as opposed to MEG. For example, EEG is more sensitive to radial electrical fields generated by activity on the gyral crests, while MEG is more sensitive to radial magnetic fields (resulting from orthogonal electrical fields) from activity in the sulcal banks (Ahlfors et al., 2010). When ECoG is recorded from subdural electrodes, it is also preferentially sensitive to activity from gyral crests. When recorded from depth electrodes, however, this depends on the precise location of the contacts.

The distance between the cerebral cortex and scalp-electrodes (EEG) or magnetometers (MEG) makes these signals less sensitive to cortical activity at higher frequencies (> 40 Hz) compared to ECoG due to out-of-phase mixing of higher frequency activity arriving at the sensor with different path lengths (Pfurtscheller and Cooper, 1975).

While both EEG and MEG signals can be studied at the level of scalp electrodes or magnetic sensors, attribution of signal features to brain anatomy requires source modeling. This is typically not necessary in the case of ECoG signals since the recording electrodes are immediately adjacent to the cerebral cortex. Scalp potentials that are recorded as EEG require the propagation of cortically generated signals through the meninges, cerebrospinal fluid, skull, and scalp. Modeling the sources of these signals onto the cortex therefore requires detailed head-modeling that accounts for the properties of these extracerebral elements in addition to brain anatomy. Magnetic fields generated by the cortex are not significantly influenced by the properties of extracerebral tissue, however. Modeling the sources of neuromagnetic activity recorded by an array of MEG sensors is therefore more straightforward.

Since the 1980s, many studies have established certain key characteristics of EEG responses to sensory input and cognitive processing: local engagement of the cerebral cortex in response to a sensory stimulus or task produces an increase in high-frequency power in the gamma band (> 40 Hz), often referred to in the literature as event related synchrony (ERS), which is simultaneously accompanied by a decrease of power in the alpha (8–12 Hz) and beta (13–30 Hz) bands, referred to as event-related desynchronization (ERD) (Pfurtscheller, 1991, 2001; Crone et al., 1998, 2006, 2009; Pfurtscheller and Da Silva, 1999; Tallon-Baudry and Bertrand, 1999). Functional mapping based on these EEG phenomena have been extensively studied using both intraoperative ECoG recordings during tumor surgery (Edwards et al., 2009) as well as long-term recordings from subdural or depth electrodes in patients being evaluated for epilepsy surgery (Sinai et al., 2005; Crone et al., 2009; Arya et al., 2018). The extent to which task or resting state functional connectivity, in different frequency bands, provides non-duplicative information about function localization, [compared to task-related high-gamma activity, event related potentials (ERPs), or DES], remains to be determined.

Compared to traditional surface EEG, ECoG possesses excellent signal-to-noise ratio, which is especially important for measuring high-gamma band (∼ ≥ 70 Hz) activity. High gamma, specifically, is believed to represent the local synchronized activation of neurons (Ray et al., 2008). As such, high-gamma is critical to assessing large-scale network communications. Like DES, the primary limitation of ECoG is the need for invasive access and patient-specific indications for placement, as well as data commonly being obtained from epileptic or lesional brains, as that is where clinical indications allow (Sadaghiani et al., 2022). From the ECoG waveform or prespecified frequency bands, 3 features (*amplitude/power, coherence, and cross-frequency coupling*) are commonly examined. Numerous potential amplitude/power biomarkers exist for a variety of physiological properties. As previously noted, high-gamma (≥ 70 Hz) is thought to be associated with local synchronized neuronal firing. For example, ECoG gamma band activity increases prior to finger movement onset in the pre-motor and somatosensory areas and with movement onset in the motor cortex (Sun et al., 2015). Other frequency bands (i.e., μ and β) tend to decrease with movement onset, remain suppressed during the task, and may be associated with gross characteristics such as grip aperture (Flint et al., 2014, 2017; Sun et al., 2015). Likewise, high-gamma activity is increased in the inferior frontal gyrus and temporal lobe during speech production and auditory processing (Chang et al., 2013). Because of the task-specific nature, regional specificity, and temporal scale, ECoG has been used as the source of brain-machine interface decoding in several applications (Krucoff et al., 2016; Flint et al., 2017; Aabedi et al., 2021). For example, motor cortex gamma power has been shown to account for ∼30–40% of the variance for finger force output and ∼12% of variance of precise finger movements (Flint et al., 2014, 2017), both of which increase when other features are included. Accuracy of ECoG decoding is limited, unfortunately, by limited spatial exposure on the upper end and large electrode size/lack of single cellular data on the smaller end (Krucoff et al., 2019).

In addition to frequency-specific power/amplitude informing behavioral output (e.g., motor action), more recent ECoG analyses have focused on functional interconnectedness of brain foci often measured as *interelectrode coherence*. Relevant to motor behaviors, gamma band inter-electrode coherence increases in the pre-frontal cortex prior to movement onset. This corresponds to the large negative cortical readiness potential occurring prior to movement onset (Kim et al., 2017). Additionally, inferior frontal gyrus and dorsal pre-motor cortex were shown to be functionally connected (via ECoG coherence) to the temporal lobe during both speech vocalization and auditory processing with speech moreso than audition (Kingyon et al., 2015).

**FIGURE 5:**
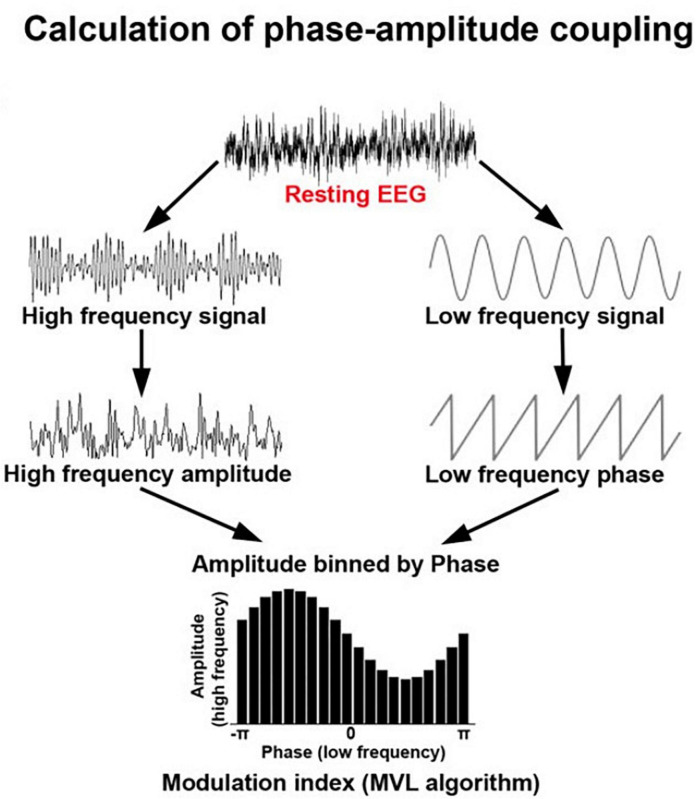
Schematic showing the calculation of phase-amplitude coupling which is commonly used to assess dynamic integration of information in the brain. Adapted from Rustamov et al. (2022) under creative commons license.

*Cross-frequency coupling* (Figure 5) describes the phenomenon where higher frequencies are nested inside lower carrier frequencies (Pagnotta et al., 2020; Rustamov et al., 2022). This can take the form of phase-amplitude coupling, phase-phase coupling, or amplitude-amplitude coupling (Jensen and Colgin, 2007). In phase-amplitude coupling, for example, the amplitude of the high frequency content is nested within the phase pattern of a lower frequency band (Abubaker et al., 2021). For example, high gamma amplitude may be highest when the phase of a theta rhythm is at π/2 and lowest when the theta rhythm is at 3π/2. Cross-frequency coupling is thought to be associated with coordination of local and large-scale network oscillations (Abubaker et al., 2021).

### 3.3. Magnetic resonance imaging

#### 3.3.1. Tractography

Diffusion weighted tractography (i.e., tractography) of the brain has been a tremendous resource for non-invasively imaging cortical white matter and its directionality (Yang J. et al., 2021). Diffusion weighted imaging involves collecting at least one imaging volume with a static magnetic field (termed b_0_) along with at least one 1 volume with an applied diffusion gradient (i.e., b_1000_) (Yang J. et al., 2021). The b_1000_ image is commonly used to diagnose strokes (Osa García et al., 2022). For the assessment of large-scale networks, larger numbers of volumes need to be obtained through applying at least 6 vectors of diffusion gradients to obtain a diffusion tensor image (DTI) (Drake-Pérez et al., 2018; Doyen et al., 2022). This allows deterministic fiber tracking to be conducted (Drake-Pérez et al., 2018). Obtaining many more vectors allows more sophisticated models (such as constrained spherical deconvolution) to be applied from which either a deterministic or probabilistic model of fiber tracks may be obtained (Doyen et al., 2022). In general, the more diffusion vectors applied during the scan, the more precise fiber tracking will be obtained (Doyen et al., 2022). Additionally, other more sophisticated models rely on applying multiple “shells” of diffusion gradients (i.e., B_1000_, B_2000_, B_3000_, etc.) (Van Essen et al., 2013). For example, collecting multiple low B values (i.e., ≤ B_1000_) allows for modeling the both free-water and a neurally constrained diffusion tensor within a single voxel (Nemmi et al., 2022). Additionally, collecting a wide range of diffusion gradients up to B_6000_ (Olson et al., 2019) allows for the application of a mean apparent propagator model which has been shown to better estimate tissue microstructure than diffusion tensor models (Özarslan et al., 2013; Avram et al., 2016). Importantly for clinical feasibility, scan time for mean apparent propagator models have been optimized for collection in < 10 min (Olson et al., 2019). Strengths of this technique include whole brain coverage and visualization and quantification of white matter pathway anatomy. Weaknesses include loss of validity in edema-present voxels using standard models (i.e., diffusion tensor).

### 3.3.2. Functional MRI

Functional MRI (fMRI) images the blood oxygen level dependent (BOLD) hemodynamic response to neural activity (Biswal et al., 1995). fMRI has become ubiquitous in tertiary care centers as a non-invasive marker of large-scale network topography and, more recently, connectivity. The utility of fMRI rests in the physiological coupling between neuronal electrical activity, cellular metabolism, and blood flow (Agarwal et al., 2021). That is, neuronal depolarizations necessitate oxygen delivery which increases local blood flow. Therefore, fMRI indirectly measures brain activity via fluctuations in deoxygenated blood (Agarwal et al., 2021). Conventionally, sampling the whole brain requires ∼2–3 s (0.33–0.5 Hz) to complete; however, some technological advances have allowed whole-brain sampling as quickly as ∼0.72 s (1.39 Hz) or lower as in the Human Connectome Project (Constable and Spencer, 2001; Van Essen et al., 2013). This limitation in sampling rate is obviated by the slowness of the hemodynamic response, as the peak power in the hemodynamic signal is below 0.1 Hz (Biswal et al., 1995).

Studies examining large-scale networks using fMRI typically employ a resting-state or task-based design or combine these approaches (Van Essen et al., 2013; Cole et al., 2021; Vinehout et al., 2022). In the resting-state, several canonical networks (i.e., default mode, dorsal attention, sensorimotor, etc.) are readily identifiable (Smith et al., 2009; Schaefer et al., 2018). During tasks, connectivity within networks necessary for completing the task (i.e., “task-positive” networks) demonstrate increased connectivity relative to rest, whereas networks unrelated to the task demonstrate decreased connectivity (Kalinosky et al., 2019; Cole et al., 2021; Vinehout et al., 2022).

## 4. Applications of network and mapping techniques to brain tumor surgery

As network neuroscience advances, peri-operative mapping for brain tumor resections is also evolving (Duffau, 2021). The following section examines how these techniques are being used to impact brain tumor resections.

### 4.1. Electrophysiological techniques

Since Dr. Penfield, DES has been used in standard clinical practice to map positive and negative responses across cortical and subcortical areas (Aaronson et al., 2021). One group with extensive experience in this area has generated a probabilistic atlas of numerous functional perturbations across sensorimotor and language domains throughout the brain (Sarubbo et al., 2020). Importantly, while this work has often confirmed the conventional motor and sensory distributions in the pre- and post-central gyri, respectively, it did also identify some reciprocity more recently (thought to be indicative of U-shaped fibers) (Sarubbo et al., 2020), and newer techniques suggest that our knowledge of the somatotopy of the motor homunculus may be incomplete (Jensen et al., 2022; Gordon et al., 2023).

Focal slow rhythms in EEG have been recognized with brain tumors and other structural lesions from the earliest days of EEG. In 1936 using an array of just 3 scalp electrodes, Walter (1936) showed the presence of focal slow waves that he named “delta” in the vicinity of brain tumors. Subsequent studies determined that slow activity in the delta (0.5 to 4.0 Hz) and theta (4 to 8 Hz) frequency ranges can be seen in association with a variety of conditions including tumors, traumatic brain injury, degenerative disorders, hypoxic states, and metabolic disturbances (Joynt et al., 1965; Fischer-Williams and Dike, 2005). Power-ratio analyses indicate that the increase of low-frequency power around tumors is typically also associated with a loss of high-frequency power (Nagata et al., 1985).

A few recent studies have applied functional-connectivity estimation methods to study peritumoral networks with EEG. Higher pre-operative local and global functional connectivity in patients with low-grade gliomas or meningiomas in the language-dominant hemisphere appears to predict worse language outcomes on 1-year follow-up (Wolthuis et al., 2021). Although changes in EEG power across frequency bands tend to be most salient in the vicinity of brain tumors, a recent study found that cognition in patients with left frontal gliomas was negatively correlated with beta frequency band connectivity between the contralateral (right) anterior prefrontal and parietal lobules (Ueda et al., 2022). Remote changes in functional connectivity may seem puzzling at first glance, but there is evidence supporting the notion that increased responses and enhanced functional connectivity in regions far from the tumor location may be related to functional compensatory mechanisms (Yang et al., 2022).

In the context of tumor surgery, ECoG has historically been used to identify epileptogenic peritumoral areas or monitor for after-discharges during during electrical stimulation mapping around the tumor (Yang et al., 2014). Intraoperative ECoG recordings can identify epileptic peritumoral networks based on interictal biomarkers of epilepsy such as epileptic spikes and high frequency oscillations (Berger et al., 1993; Mikuni et al., 2006; Yao et al., 2017; Feyissa et al., 2018). Interestingly, in patients with brain tumor-related epilepsy, (Feyissa et al., 2018) high frequency oscillations (i.e., > 80 Hz epileptic associated oscillations) are associated with neuroinflammation (Sun et al., 2022) and a variety of tumor molecular pathways (Hills et al., 2022). Additionally, increased high frequency oscillations have been associated with IDH-wildtype status, and overall survival is associated with greater surface EEG excitability (Feyissa et al., 2018; Tobochnik et al., 2022). While not fully understood, tumor-related epilepsy (and thus high frequency oscillations) is thought to be related to IDH-related increases in glutamate and/or D-2-hydroxyglutarate and reduced GABAergic inhibition (Armstrong et al., 2016; Chen et al., 2017). Thus, ECoG assessments of high frequency oscillations may be a critical marker of length of survival in patients with brain tumors.

As with scalp EEG, the association between abnormal low frequency neuromagnetic activity and brain tumors is well recognized (Baayen et al., 2003; de Jongh et al., 2003). Tumor-related focal delta activity in the peritumoral cortex has also been noted to be greater in the case of intra-axial tumors that involved subcortical fibers than for extra-axial tumors, and patients with an increased delta activity exhibited poor recovery of function, at least within the early postoperative period (Oshino et al., 2007). As observed in scalp EEG studies, a decrease in power in the high-alpha and beta frequencies has also been reported in peritumoral regions (Shekoohishooli et al., 2021).

There is vast literature on the use of MEG for localizing the sources of epileptic spike activity in patients with drug-resistant epilepsy, but few have focused specifically on peritumoral epilepsy (Patt et al., 2000; Birbilis et al., 2014). In a study that correlated tumor type with the location of source dipoles, sources were noted to be significantly closer to the tumor border in glial tumors compared to mixed glial-neuronal neoplasms or metastatic tumors, although tumor cells did not appear to be directly involved in generating spikes (Patt et al., 2000). Similarly, although MEG is an approved tool for the presurgical mapping of motor and primary sensory cortex and for determination of hemispheric language dominance in patients with epilepsy, brain tumors, and cerebral vascular lesions, the existing literature specific to functional mapping in brain tumors is sparse. One of these studies found that at a 10-mm distance-threshold, MEG showed a greater specificity than fMRI for both motor and language mapping but a lower sensitivity for motor mapping (Ellis et al., 2020). Several studies of language mapping have claimed good accuracy with MEG in localizing Broca’s and Wernicke’s areas in brain tumor patients using a variety of source modeling approaches and tasks with significant overlap with fMRI activations in some of these studies (Grummich et al., 2006; Pang et al., 2011; Huang et al., 2016).

Over the last couple of decades there have been many studies of functional connectivity based on resting state MEG source dynamics in patients with brain tumors. The studies examine both the pathophysiology of cognitive impairment in this population and clinical applications such as identifying high functional connectivity (HFC) nodes around tumors. An early study of broad band (0.5–60 Hz) and gamma band (30–60 Hz) MEG functional connectivity performed at the sensor level, suggested loss of functional connectivity in multiple regions with left hemispheric tumors registering a greater impact (Bartolomei et al., 2006). Subsequent studies that have looked at multiple frequency bands consistently show evidence of increased theta-band connectivity both at short and long distances from tumor locations (Bosma et al., 2008, 2009; Derks et al., 2014, 2021). These studies also show significant correlations between increased theta-band connectivity and degree of cognitive dysfunction associated with low-grade gliomas. This suggests that cognitive dysfunction in these patients may be related to widespread changes in the strength and spatial organization of functional brain networks. Increased theta-band connectivity also appears to correlate with increased seizure frequency in patients with epilepsy related to low-grade gliomas (Douw et al., 2010; van Dellen et al., 2012). Furthermore, while gliomas tend to occur more often in brain regions that have high network-clustering, they have greater detrimental effect on whole-brain networks and cognition when located in regions with low-clustering (Derks et al., 2021).

From the perspective of immediate clinical applications, several studies have now demonstrated that preservation of peritumoral areas with HFC in the alpha band (8–12 Hz) based on resting state MEG can predict post-operative neurological outcomes (Guggisberg et al., 2008; Martino et al., 2011; Tarapore et al., 2012; Lee et al., 2020). Such HFC nodes also have a high probability of yielding positive responses during intraoperative electrical stimulation mapping of sensory, motor, and language functions, and, quite remarkably, a 100% negative predictive value for the absence of eloquent cortex during stimulation mapping (Martino et al., 2011).

Magnetoencephalography (MEG) has also been used by a few groups to study the reorganization of cortical areas that support motor and language function around brain tumors before and after surgical resections, especially in patients with recurrent tumors. Detectable shifts in the location of motor representations that correlated with a frontoparietal location of the tumor, presence of motor deficits, and interval between the scans have been reported (Bulubas et al., 2020). Likewise, laterality indices of hemispheric language dominance were noted to change in a majority of patients with recurrent gliomas, with the scale and direction of change depending on initial degree of lateralization and tumor location (Traut et al., 2019). Enhancement of post-operative alpha-band connectivity has been observed in peritumoral regions in all patients regardless of the low grade glioma location and has been suggested as a marker for post-operative plasticity (Lizarazu et al., 2020). After glioma resections, an increase in alpha-band connectivity compared to the pre-operative baseline between the default-mode network areas was found to correlate with improvement in post-operative verbal memory, while an increase in alpha-band connectivity across the frontoparietal areas correlated with improvement in attention (Van Dellen et al., 2013).

### 4.2. MRI-based techniques

**FIGURE 6:**
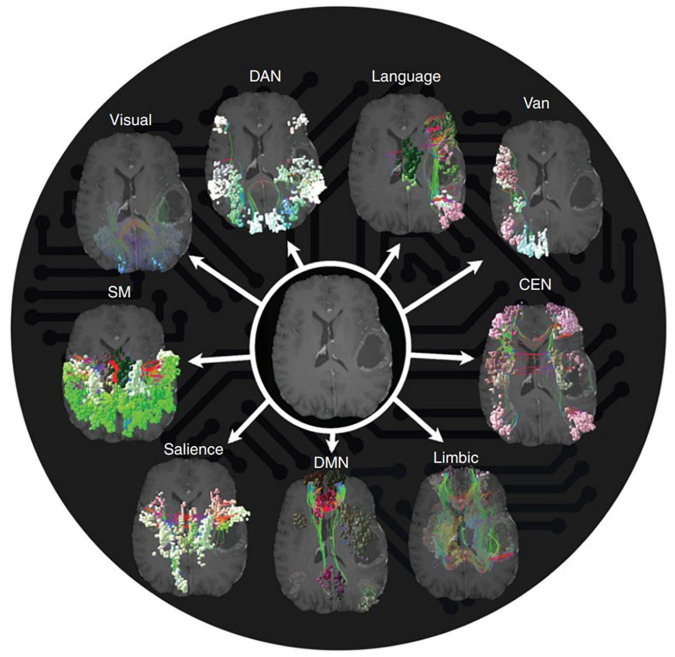
Individual large scale network fiber tracking using Quicktome software. Reproduced with permission from Morell et al. (2022), all rights reserved.

Tractography is now used routinely in many centers to identify the relationship of brain tumors to critical white matter tracts (Figure 6). More recently, researchers have used tractography to examine structural “disconnectomes,” or the probability that a lesion will interrupt the structural connections between paired regions. Similarly, recent work has demonstrated that, in patients with glioblastoma, both structural connectivity is decreased relative to controls and reductions of long range connections is associated with poorer overall survival (Wei et al., 2022).

**FIGURE 7:**
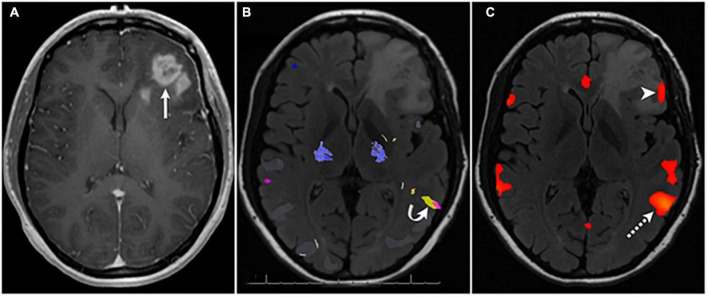
Seed based correlation analysis localizes language network [panel **(C)**] after failure of task-based fMRI [panel **(B)**] to identify language activation in Broca’s area in a patient with left frontal glioblastoma [panel **(A)**]. Used under creative commons license from Kumar et al. (2020).

Similarly, resting-state fMRI has been frequently used to define the topography of networks associated with language and motor behavior (Hacker et al., 2019; Kumar et al., 2020; Mitchell et al., 2020; Daniel et al., 2021; Yang P. et al., 2021). Commonly, these studies employ seed-based correlation (Figure 7) analyses to define the boundaries of these networks for the purpose of surgical planning (Hacker et al., 2019). The major benefit of resting-state fMRI for defining network boundaries is its independence from task performance in the scanner. This is important in participants with poor ability to perform the desired task or for networks poorly defined by a task paradigm (such as the salience network). While clinically useful in determining the hemispheric laterality of language dominance and general localization of motor, speech, and language areas, fMRI does not provide as assessment of the critical or hierarchical nature of BOLD-positive sites. In other words, areas that light up with BOLD may not result in a neurological deficit if injured, meaning that, while they are active during a task, they are not necessary for its performance (i.e., redundant). Additionally, pure motor information can be difficult to isolate from sensation during a motor task, as sensory information is almost constantly flowing. fMRI is also limited by its large temporal scale and the indirect nature of the recording; that is, fMRI measures the hemodynamic response to neural activity rather than neural activity directly. The latter can confound results in brain tumor cases when neurovascular decoupling can occur (Agarwal et al., 2021).

Functional magnetic resonance imaging (fMRI) and diffusion tractography mapping prior to surgical resection have been reviewed previously (Lee et al., 2016; Hu and Hoch, 2021; Ille and Krieg, 2021; Sparacia et al., 2021). The review by Sparacia et al. (2021) includes a clinical workflow for their institution based on readily available software. Newer research has extended these fMRI and tractography mapping techniques by using tractography to identify an individual patient specific parcellation for the brain (Doyen et al., 2022; Dadario et al., 2023). Similarly, this same group has proposed that highly connected areas (i.e., “hubs”) may identify non-speech/motor areas that should be left during surgical resection (Tanglay et al., 2023). This concept will be explored further in the “Future directions” section below.

### 4.3. Applications to pediatric brain surgery

Pediatric neurosurgery patients pose unique challenges for many reasons, some of which include their inability to participate in complex tasks and an actively developing brain. Unfortunately, the pediatric-specific literature on brain mapping techniques is limited and largely focused on epilepsy (Coppola et al., 2016; Chou et al., 2018). Presurgical planning with fMRI can pose several challenges, including inability to stay still in the scanner and lack of cooperation with task performance (Maheshwari, 2019). In younger children and non-cooperative patients, passive sensorimotor mapping under sedation may be an option (Maheshwari, 2019). High concurrence of the cortical activation between active and passive motor tasks has been demonstrated in adults (Kocak et al., 2009). Comparison of passive motor mapping under sedation with active motor mapping and direct cortical stimulation has been reported in children with focal brain lesions, masses, or cortical dysplasia (Ogg et al., 2009).

Rosazza et al. (2014) assessed the degree of correlation between sensorimotor mapping with task-based and rs-fMRI techniques. While larger areas of activation were seen with rs-fMRI, they concluded there was enough agreement between the techniques to warrant its use in patients who were unable to perform the tasks. Similarly, several studies comparing language localization between task-based fMRI and rs-fMRI in adult patients found good concordance between task-related language activation and resting-state language networks (Branco et al., 2016; Sair et al., 2016; Maheshwari, 2019). Given these results, rs-fMRI is particularly appealing for the evaluation of whole brain networks in children.

The pediatric brain tumor population is also unique in that nearly two-thirds of pediatric brain tumor patients are diagnosed with an infratentorial brain tumor. The most frequent infratentorial or posterior fossa tumors (PFTs) are medulloblastomas (40%), astrocytomas (30%), and ependymomas (10%) (Baudou et al., 2021; Sleurs et al., 2021). Their treatment depends largely on tumor histology and the patient’s age (Baudou et al., 2021). Many of these patients suffer long-term effects secondary to direct damage from the tumor, damage from surgery, and/or toxicity from adjuvant therapy such as chemotherapy and radiotherapy. Young children with posterior fossa brain tumors are particularly at risk for the development of cerebellar mutism syndrome (CMS), which may present with key symptoms including transient muteness/reduced speech, ataxia, hypotonia, dysphagia, cranial nerve impairment, and emotional lability. Its onset can be delayed and recovery is prolonged and not always complete. Previously thought to be responsible only for balance and coordination, a newer body of evidence has revealed the cerebellum also plays a central role in working memory and procedural memory, both of which are involved in motor and cognitive learning (Baudou et al., 2021). The constellation of symptoms in CMS, including its similarities to other syndromes arising from topographically distinct regions such as the supplementary motor area (SMA) involved in SMA syndrome, have raised theories on the existence of interrelated neural loops supporting a range of higher functions (Grønbæk et al., 2020).

Recent studies have used lesion-symptom mapping to create a cerebellar white matter atlas that shows cerebello-thalamo-cerebral outflow tracts in the superior cerebellar peduncle (SCP) next to the dentate nucleus (DN) and inferior vermis are associated with impaired motor, cognitive, executive, and behavioral function (Grosse et al., 2021).

Miller et al. (2010) demonstrated decreased cortical perfusion in association with decreased cerebellar perfusion in patients with CMS. Similarly, decreased metabolism in the right cerebellar and left frontal lobes has been detected on fluorodeoxyglucose positron emission tomography/computer tomography in a patient with CMS (Gedik et al., 2017). Furthermore, functional connectivity studies have demonstrated connectivity of the dentate nuclei with SMA and Broca’s areas, further strengthening the hypothesis of dysfunctional overlapping pathways or networks in PFT survivors (Grønbæk et al., 2020). Language processing is complex and may differ from classical language areas in the setting of anatomic anomalies (e.g., cortical dysplasia, tumor invasion, etc.), abnormal electrical circuitry (e.g., epilepsy), or as a response to brain injury, particularly in the neuroplastic pediatric brain (Vadivelu et al., 2013; Chou et al., 2018).

Sleurs et al. (2021) reported widespread microstructural changes in supratentorial brain areas in addition to macrostructural alterations in infratentorial brain areas in survivors of PFTs. When comparing all brain regions (nodes) in these patients compared to healthy controls, significant differences in nodal strength was seen in “hubs” or regions that were most densely connected. Furthermore, these highly connected nodes were most affected in CNS tumor survivors and highly correlated with intelligence scores (Sleurs et al., 2021).

While gross total resection of posterior fossa tumors contributes to relapse-free survival (Grosse et al., 2021), preventing injury to the SCP and deep cerebellar nuclei is critical for preserving optimal motor and cognitive function in pediatric cerebellar tumor survivors. Multimodal perioperative imaging aimed at evaluating structural and functional alterations with high spatial and temporal resolution (e.g., tractography, MEG, task-based fMRI and/or rs-fMRI, with regional network and cross-network connectome analysis) may help identify imaging biomarkers in high-risk patients. These imaging biomarkers may then allow physicians to individualize treatments, avoid complications, and ultimately improve the recovery and long-term outcomes of these patients.

### 4.4. Brain tumor-related cognitive dysfunction and depression

A decline in cognitive function and the emergence of depression can have similar and compounding effects on brain tumor patients. Either symptom can diminish independence, hinder social relationships, and/or negatively impact overall perceived quality of life (Noll et al., 2017; Lange et al., 2019). Coping and adjustment related syndromes may also occur after diagnosis, during treatments, or during survivorship (Noll et al., 2019). Some estimate depression rates in glioma patients to be around 15–20% (Rooney et al., 2014), and up to about 90% show select features of depression (Noll et al., 2019). Not only is an individual’s depressive status important to know for quality of life purposes, but it may also help to inform prognosis, as depression has been associated with poorer overall survival in gliomas (Noll et al., 2019).

In addition to the psychological manifestation of these symptoms, brain tumors themselves may directly impact emotional regulation, personality, and behavior when dysregulating their associated networks. For example, lesions in the dorsolateral prefrontal cortex typically result in a *primary executive dysfunction syndrome* (e.g., deficits in problem solving and reasoning), while lesions in orbital frontal regions can result in increased disinhibition, impulsivity, and inappropriateness. Similarly, lesions in the medial frontal region can result in *apathy* and *abulia* (Filley and Kleinschmidt-DeMasters, 1995; Cummings and Mega, 2003; Boele et al., 2015). The third syndrome, *medial frontal syndrome*, is most likely to be mis-interpreted as “depression” given the overlap in some of the key behavioral changes, such as social withdraw, emotional blunting, and disengagement.

Lesions outside of the frontal lobes may also result in changes in mood and emotional processing, such as those that disrupt parietal integration regions and temporal paralimbic structures (Boele et al., 2015). A voxel-based lesion-symptom mapping study examined mood and emotional perception as well as higher level emotional reasoning and abstraction in patients with frontal, parietal, and temporal tumors (Campanella et al., 2014). They found basic emotional interpretation was affected by temporo-insular lesions, emotion recognition was maximally impacted by anterior temporal and amygdala lesions, and higher-level emotional reasoning was maximally affected by prefrontal lesions (Campanella et al., 2014).

### 4.5. Future directions

An intriguing emerging notion is that “centrality/hubness” (i.e., the graph theory idea that an area with many strong connections is more critical than an area with fewer connections) may provide a marker of resectability beyond the boundaries of historically “eloquent” tissue (Tanglay et al., 2023). For example, an area in the superior frontal lobe (Ahsan et al., 2020) that has a number of important connections may not be suitable for resection in a given person. Conversely, if another person has a portion of “eloquent” cortex with low centrality, that could hypothetically be resectable in that individual (Ahsan et al., 2020; Yeung et al., 2021). Importantly, while tractography has been used to assign “connectivity” and subsequently “centrality” to certain nodes, many of the techniques in this review may expand upon these assessments. For example in patients with epilepsy, eigenvector centrality calculated from spontaneous ECoG was found to correlate directly with spiking at an electrode (Zweiphenning et al., 2016). This has been useful in identifying seizure onset zones (Burns et al., 2014). Likewise, centrality of a given cortical node may prove beneficial in isolating resectable boundaries. It may also be possible to apply this concept to map nodes critical for higher cognitive functions.

**FIGURE 8:**
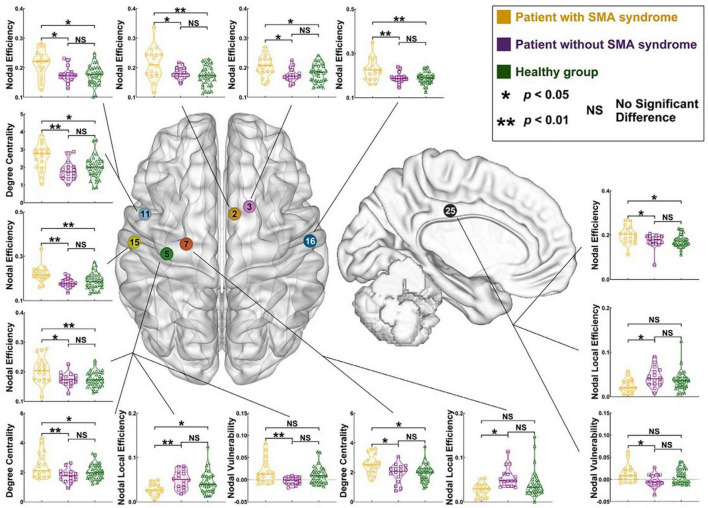
Differences in pre-operative node-level connectivity properties in patients with left hemisphere glioma with and without supplementary motor area syndrome versus a healthy cohort. Note frequently elevated nodal efficiency, degree centrality, and nodal vulnerability in the cohort with SMA syndrome. Reproduced under creative commons license from Fang et al. (2022).

One example of a well-studied temporary motor phenomenon is supplementary motor area syndrome (SMA syndrome), or when resection of the SMA in the posterior superior frontal gyrus results in acute impairments in motor control and speech (Palmisciano et al., 2022). Most often, symptoms are temporary with mean duration ∼45 days (∼80% of cases), but it can be clinically difficult to differentiate from true hemiparesis and mutism (Palmisciano et al., 2022). Persistent SMA syndrome occurs in ∼20% of reported cases (Palmisciano et al., 2022). As intraoperatively monitoring motor evoked potentials (MEPs) incompletely predicts persistent motor impairments (Giampiccolo et al., 2021a), a network approach may be able to predict patients likely to have persistent motor deficits for reasons other than primary motor tract integrity. Because it is known that the contralateral SMA is critical to the recovery from the syndrome (Chivukula et al., 2018), higher interhemispheric connectivity between the respective SMAs may be protective from persistent deficits (Young et al., 2021). It is possible that the pre-resection strength of connectivity from the SMA to the motor cortex will be greater in those who have persistent symptoms (Figure 8). Alternatively, it is possible that diffusion tractography will identify a greater number of descending streamlines directly from the SMA to the corticospinal tract in those with persistent motor impairments. Ultimately, such investigations have the potential to guide pre- and post-operative rehabilitation strategies to facilitate more rapid resolution of SMA syndrome, and/or to better predict which individuals will have persistent deficits.

Likewise, connectomic techniques may soon enable the mapping, monitoring, and protection of higher-order cognitive functions. Notably, there is a paucity of studies systematically mapping connections between nodes with high fidelity functional and health related outcome measures for many cognitive processes.

Additionally, there is untapped potential in combined mapping techniques. For example, concurrent TMS-fMRI (Bergmann et al., 2021) or TMS-EEG (Tremblay et al., 2019) have, to our knowledge, never been examined in patients with brain tumors during pre-resection mapping. Concurrent TMS-fMRI and TMS-EEG involve interleaving TMS stimulations into the fMRI and EEG recording paradigms. Techniques such as TMS-fMRI could solve some of the issues with pre-surgical mapping with task-based fMRI (i.e., motivation, functional capacity, understanding directions, etc.). That is, measuring BOLD responses elicited by TMS stimulation would be task-independent, similar to resting state fMRI mapping, but could also alleviate the computational difficulties of resting-state fMRI (i.e., seed-based vs. independent component analysis-based techniques). However, this would require specialist trained staff competent on both techniques (Bergmann et al., 2021).

## 5. Advancing neuroscience through neurosurgical oncology

**FIGURE 9:**
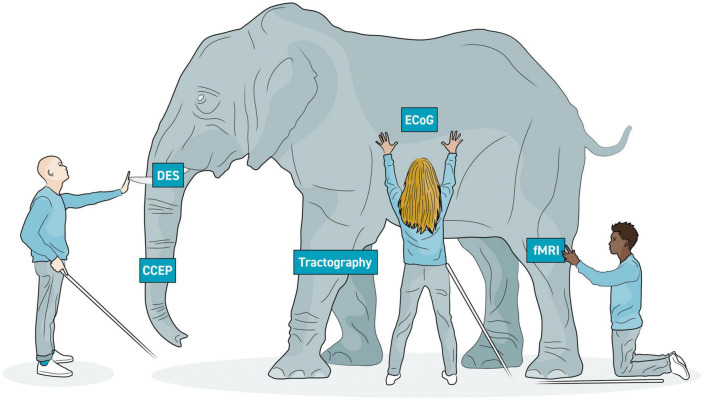
Elephant model of brain imaging modalities. In the parable of the blind men and the elephant, several blind men are attempting to learn about an elephant by touching it. However, they all touch it in different places and draw different conclusions. Similarly, the various neuroimaging modalities each allow us to draw partial conclusions of brain structure and function. A more complete understanding of the brain will be more possible with multi-modal imaging. Each modality is able to image one aspect of respective large-scale networks.

Using the limited available methods to understand large-scale brain networks is characteristically like the parable of the blind men and the elephant: while each technique has been developed to examine the “elephant” from various vantage points, none is able to achieve the whole picture on its own (Figure 9). Here, of course, the elephant is brain function while each blind man is a single, limited mapping technique. Further complicating matters is the reality that neurological networks simultaneously exist on micro- and macroscopic scales, making obtaining a comprehensive understanding of the CNS virtually impossible (Krucoff et al., 2016, 2019).

Invasive electrophysiological recording (ECoG) and stimulation evoked methods (e.g., DES and CCEP) have excellent signal fidelity to identify topography and connectivity, but they are limited by access. Imaging methods such as structural and functional MRIs provide whole brain perspective on topography and connectivity, but measurements are indirect, temporal resolution is large, and inferences about necessity, redundancy, and specificity often cannot be drawn. Nevertheless, by merging modalities into multi- or even omni-modal designs (Van Essen et al., 2013; Samuel et al., 2022), the shape of the elephant is gradually growing clearer.

Notably, work in multi-modal connectomics is ongoing. Studies have already combined CCEPs and tractography, while others have combined DES with tractography and resting state fMRI (Silverstein et al., 2020). Brain tumors present a unique opportunity to probe these networks because many tumors, especially metastases, cause focal network disruption with measurable deficits that can be tracked after resection and recovery. Additionally, multimodal data is often gathered as part of pre- and intraoperative standard of care. For example, with lesions near language and motor systems, tractography and fMRI are routinely obtained. DES mapping is frequently done intra-operatively as well. ECoG is commonly obtained to monitor for seizures. As it stands, merging these modalities has the potential to advance our understanding of communication within and between the intrinsic cortical networks. Moreover, pairing these modalities with task performance (Conway et al., 2023; Taquet et al., 2023) can assess the processes of task execution, facilitation, and inhibition. The combinations of DES and task performance have been used by Rossi et al. (2021) to aid in motor mapping by having patients manipulate an object while quantifying electromyography. This has been used to define frontal and parietal lobe topographical boundaries of clumsiness, inhibition, and facilitation (Fornia et al., 2020, 2022; Simone et al., 2020; Viganò et al., 2022). Within this realm, our lab has ongoing efforts to synchronize multiple recording devices in the operating room to assess networks important for several facets of hand motor control (Taquet et al., 2023).

Identification of markers of large-scale network dysfunction may lead to new therapeutic targets for rehabilitation in patients with brain tumors. For example, transcranial magnetic stimulation (TMS) combined with exercise may be a beneficial treatment in patients with tumor and radiation-related motor impairments (Tang et al., 2022). Additionally, connectomics may facilitate improved outcomes assessments for therapies such as cognitive rehabilitation. Thus, imaging large-scale brain networks may help improve survivorship care (Leeper et al., 2022).

**FIGURE 10:**
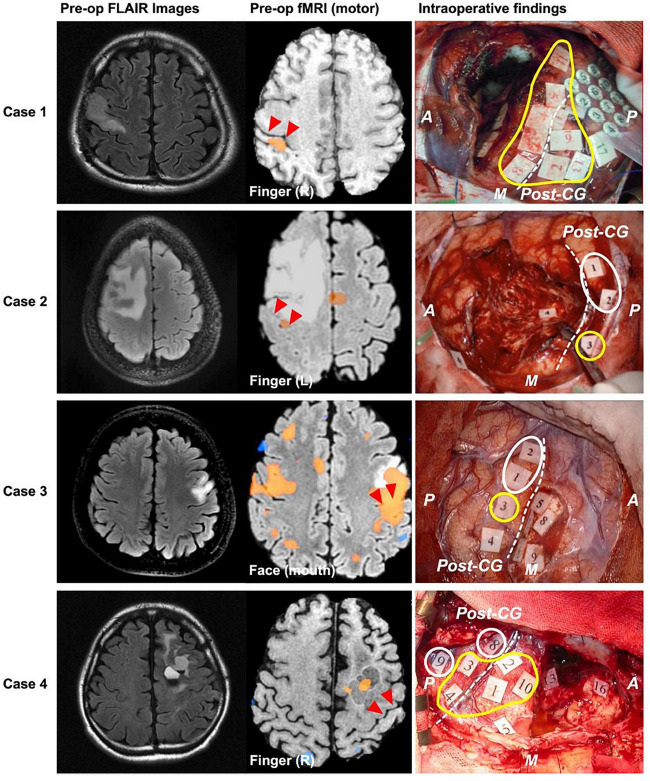
Examples of cases where motor function relocated to posterior to the central sulcus in response to brain tumor. **Left**, **middle**, and **right** columns show preoperative FLAIR, preoperative fMRI BOLD results, and intraoperative mapping results, respectively. Red triangles and white dashed lines in the **middle** and **right** columns, respectively indicate central sulcus. Yellow and white circles indicate positive mapping results for motor and sensory, respectively. A, anterior; P, posterior; M, medial; CG, central gyrus. Redistributed from Nakajima et al. (2020) under creative commons license.

Also critical is the concept of cortical topological plasticity. Previous work has demonstrated that cortical representations of language and motor function may be relocated either by natural processes such as tumor invasion (Duffau et al., 2000; Nakajima et al., 2020) (Figure 10) or stimulation-based prehabilitation (Rivera-Rivera et al., 2017). Invasive and non-invasive multimodal brain imaging (e.g., DES, ECoG, CCEPs, tractography, fMRI, etc.) will be critical to identifying anatomical boundaries of and potential mechanisms for such plasticity. Understanding the brain’s ability to adapt around injury, the boundaries of its plasticity, and how we can modulate it to promote functional recovery represents a frontier of neurological surgery (Hamer and Yeo, 2022).

## 6. Conclusion

Because of the central nervous system’s seemingly endless complexity across immense size, spatial, and temporal scales, attaining a comprehensive understanding of all its states seems virtually impossible. However, gaining enough of an understanding to help better guide decision making for individuals facing brain tumor surgery is within reach. This is the focus of laboratories such as ours that are employing multi-modal connectomic imaging techniques with longitudinal behavioral studies and intraoperative stimulation/recording techniques to better understand how brain tumors perturb neural networks and how that results in neurological dysfunction, as well as how neural networks recover after surgery and what might be done to aid in their protection or reconstitution. When studied carefully, brain tumors provide a unique opportunity to test novel hypotheses about neural network function by probing networks directly and invasively with focal disruptions and monitoring how recovery occurs post-intervention. Such research may lead to improvements in understanding how neuroplasticity may be strategically modulated to improve surgical resections and functional recovery.

## Author contributions

TB: conceptualizing, drafting, revising, and final approval. PP, AB, EA-Q, and MR: drafting, revising, and final approval. MK: conceptualizing, revising, and final approval. All authors contributed to the article and approved the submitted version.
